# Toxicological Analysis of the Arylnaphthalene Lignan Justicidin B Using a *Caenorhabditis elegans* Model

**DOI:** 10.3390/molecules29235516

**Published:** 2024-11-22

**Authors:** Barbara Sciandrone, Roméo Arago Dougué Kentsop, Roberta Pensotti, Gianluca Ottolina, Iride Mascheretti, Monica Mattana, Maria Elena Regonesi

**Affiliations:** 1 Department of Biotechnologies and Biosciences, University of Milano-Bicocca, 20126 Milan, Italy; barbara.sciandrone@unimib.it (B.S.);; 2 Institute of Agricultural Biology and Biotechnology, National Research Council, 20133 Milan, Italy; romeo.dougue@ibba.cnr.it (R.A.D.K.); iride.mascheretti@cnr.it (I.M.); monica.mattana@cnr.it (M.M.); 3 Institute of Chemical Sciences and Technologies “Giulio Natta”, National Research Council, 20131 Milan, Italy; gianluca.ottolina@cnr.it; 4 Milan Center of Neuroscience (NeuroMI), 20126 Milan, Italy

**Keywords:** secondary plant metabolite, anti-cancer, toxicological study, *Caenorhabditis elegans*, justicidin B, lignans

## Abstract

The screening of plant-derived compounds with anti-cancer properties is a promising strategy to meet the growing need for new, safe and effective anti-cancer drugs. Justicidin B is a plants secondary metabolite that displays anti-cancer properties in several tumor cells. Therefore, it represents a good candidate. We used the 3R-compliant organism *Caenorhabditis elegans* to evaluate the safety of justicidin B produced by *in vitro*-grown adventitious roots of *Linum lewisii*. We showed that a dose of 100 µg/mL justicidin B does not affect worm vitality in either short-term or chronic administration; in contrast, the 200 µg/mL dose induces a lifespan reduction, but only in short-term daily treatment. We attributed this effect to its accumulation in lipofuscin granules in the pharynx as observed through confocal analysis. HPLC analysis confirmed the higher accumulation justicidin B with a 200 µg/mL dose but also revealed the presence of metabolic derivatives that could be responsible for the toxicity. We also demonstrated that the 100 µg/mL dose does not affect worm fertility or development. Our results highlight the safety of justicidin B, supporting its employment in cancer therapy, and encourage the use of a *C. elegans* model as an appropriate tool to assess compounds’ toxicity before moving to more complex organisms.

## 1. Introduction

Secondary plant metabolites (SMs) are organic substances involved in plant defense against herbivores and microorganisms and in systemic resistance under stress conditions and other environmental adversities [1]. The interest in these compounds arises from their wide application in nutritional, cosmetic, medical, pharmaceutical and agricultural fields [2,3]. Among these molecules, the lignans represent a large class of phenolic compounds characterized by a steroid-containing chemical structure and by remarkable biological activity [4].

Justicidin B is an arylnaphthalene lignan, and thanks to its pharmacological properties, it is considered a potential lead compound for the development of novel therapeutics [5]. Indeed, justicidin B displays properties such as antifungal, antiviral, antibacterial, antiparasitic, piscicidal, antiplatelet (atherosclerosis and thrombosis) and anti-inflammatory activity [6,7,8,9]. Moreover, it exerts a cytotoxic effect on chronic myeloid leukemia cells (LAMA-8 and K-562) and chronic lymphoid leukemia cells (SKW-3) [10], breast cancer cells (MCF-7) [11], human L0V0 colorectal carcinoma cells, human gastric cancer cells (BGC-823) [12] and HeLa cervical cancer cells [13], highlighting its potential use as anticancer agent.

Several studies demonstrate its anti-proliferative and pro-apoptotic activities, i.e., its ability to alter cell morphology, induce cell shrinkage and DNA fragmentation and inhibit colony formation [14]. Specifically, justicidin B exerts its cytotoxic effect by acting on specific mechanisms in different cancer cell lines, targeting definite key proteins involved in apoptosis regulation; for example, it increases the Bax/Bcl-2 ratio by enhancing Bax expression in human melanoma A375 cells, and it causes downregulation of Bcl-2, caspase-3 and PARP-1 in the HeLa cell line [13,14,15].

Despite the extensive evidence regarding the biological activity of justicidin B, to the best of our knowledge, only one *in vivo* study of its toxicity has been carried out; in that study, using an H-albino mouse model [14], different justicidin B doses were injected intraperitoneally, and after 2 weeks of application, the main physiological parameters were evaluated. At the maximum dose of 50 mg/kg (137.25 µM) of justicidin B, no physical or behavioral changes in the treated animals were observed [14]. However, to pave the way for its possible use as an anti-cancer drug, further *in vivo* studies are necessary, especially to evaluate the toxic effects of short- and long-term administration.

*Caenorhabditis elegans* represents a good experimental model that offers the opportunity to analyze different functions in an *in vivo* system that shares features with *in vitro* models, e.g., it is cost-effective and less time-consuming than other higher organisms. One feature particularly useful for toxicological studies is the possibility of easily quantifying a wide range of *C. elegans* parameters before and after treatment. This includes the ability to monitor changes in both external phenotypes and tissue morphology by taking advantage of the animals’ body transparency. Moreover, *C. elegans* genes share a high degree of conservation with human genes, including several processes and signaling pathways [16]. From a toxicological point of view, this makes *C. elegans* a powerful model in that it makes it possible to analyze not only the toxicity but also the metabolic effects of compounds. Furthermore, many cases of conservation of mode of toxic action have been observed between *C. elegans* and mammals [17].

For these reasons, this model is considered a good predictive tool for drug screening giving the opportunity to test the toxicity of a plethora of compounds in a very short time with high predictivity [18]. In fact, a previous work showed that 89% of compounds that impair egg viability in the nematode also produce developmental defects in mammals, demonstrating the predictive power of this model [19]. In addition, an extensive study reported the toxic effects in *C. elegans* of exposure to over 900 chemicals compared with ToxCast data from zebrafish, rats and rabbits [20]. The authors found 45 and 53% concordance between *C. elegans* results and those obtained from rats and rabbits, respectively, a concordance similar to that obtained between the data of the two closest rat and rabbit (58%) [20].

Furthermore, *C. elegans*’ rapid development and short lifecycle (about 3 weeks) make it particularly suitable for Developmental and Reproductive Toxicity (DART) and aging studies. Indeed, it is possible to obtain data about both short- and long-term exposure toxicity in a shorter time than in other, more complex organisms [16]. Last but not least, the employment of a *C. elegans* model in the toxicological study contributes to a reduction in the use of vertebrate models, in accordance with the 3R principles (replaced, reduced or refined), confirming *C. elegans* as a 3R-compliant *in vivo* model.

In this work, *C. elegans* was used to evaluate the safety of justicidin B obtained from *in vitro* culture of *Linum lewisii* adventitious roots and purified as previously described [21,22]. Several toxicity assays were performed to evaluate the maximum dose tolerated in both short-term and chronic treatment, the effects on the healthspan parameters (i.e., locomotion, pumping rate and oxidative stress) and the effect on worm fecundity. Moreover, HPLC and confocal microscopy analyses were performed to quantify the justicidin B uptake at different doses and to evaluate its distribution and bioaccumulation in the body of worms. Our results clearly demonstrated the safety of justicidin B even at high doses, highlighting the importance of less-than-daily administration to avoid bioaccumulation and toxicity.

## 2. Results

### 2.1. Justicidin B Toxicity Evaluation in Short-Term and Chronic Administration

**Figure 1:**
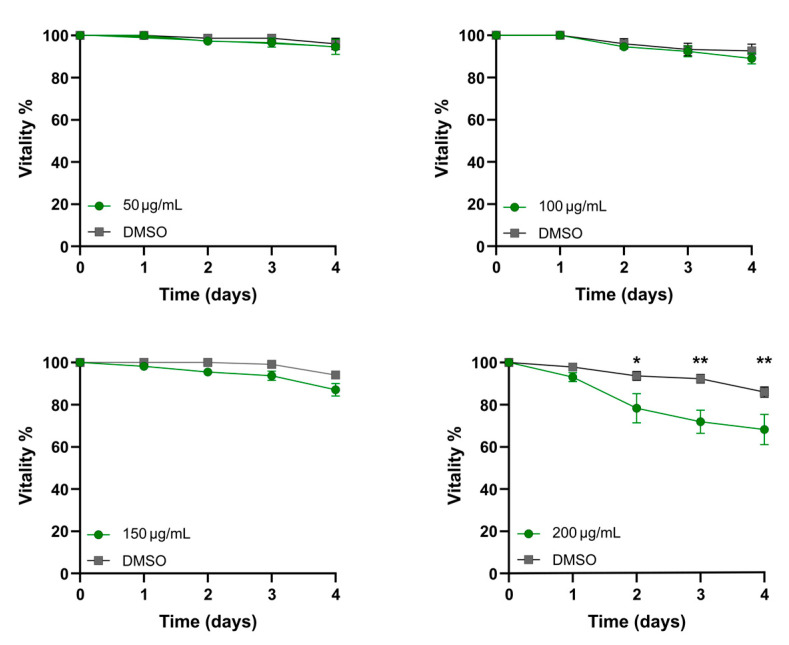
Effect of justicidin B on *C. elegans* vitality in short-term treatment. Different concentrations of justicidin B (50, 100, 150 and 200 µg/mL) were administered every day for five days to synchronized *C. elegans* populations in the absence of a food source. Graphs represent the percentage of surviving nematodes normalized to day 0. Controls were treated with DMSO in the same percentage as the corresponding justicidin B treatment. Three independent experiments were performed. Data are represented as the mean, and error bars represent S.E.M. * *p* < 0.05, ** *p* < 0.01.

Justicidin B toxicity was tested in both short-term and chronic treatment using the N2 wild-type *C. elegans* model. First, we performed a short administration, treating the worms for four days with different concentrations of justicidin B (50, 100, 150 and 200 µg/mL). This range of concentrations was been chosen by considering both the toxicity of DMSO (the biocompatible solvent in which this compound is dissolved) and the maximum tolerated amount used in a mouse model, the only *in vivo* model in which the safety of justicidin B has been assessed (50 µg/mL) [14]. Justicidin B samples and their corresponding DMSO controls were prepared in a 200 µL final volume of sterile water immediately before their deposition on the NGM plate surface. Then, 30 animals synchronized at the first day of adulthood (day 0) were moved onto NGM plates in the absence of a food source (*E. coli* OP50, heat-killed). Then, living nematodes were counted every day and moved onto a fresh plate (prepared as previously described) until the fourth day of the experiment. The results reported in Figure 1 are expressed as the percentage of animals alive normalized with respect to day 0 and showed that 100 µg/mL is the maximum dose that did not affect the worms’ vitality. In fact, an increasing toxicity of justicidin B was observed with higher doses. In particular, 200 µg/mL induced a vitality reduction of about 20% starting from day 2, and so it was selected as the toxic dose for the further analysis. 

**Figure 2:**
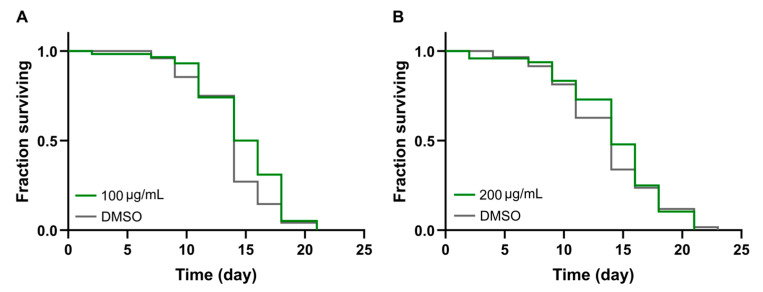
Effect of justicidin B on *C. elegans* vitality in chronic administration. Doses of 100 (**A**) and 200 (**B**) µg/mL of justicidin B were administered to a synchronized *C. elegans* population every two days in the presence of food until the whole population died. The Kaplan–Meier graphs represent the survival of the populations. Controls were treated with the same amount of DMSO contained in the corresponding justicidin B samples.

Based on these results, we analyzed the effect of 100 and 200 µg/mL justicidin B doses as a chronic treatment, consisting of repeated administrations of justicidin B or DMSO every two days from the first day of adulthood until the end of the lifespan (about 3 weeks), in the presence of a food source (*E. coli* OP50, heat-killed). The numbers of live nematodes at different days are reported as a Kaplan–Meier graph (Figure 2). It is noteworthy that, in each of the two cases, the treatment showed no significant effect on survival parameters, i.e., median and maximum lifespan (see Table 1), suggesting that less-than-daily administration of a toxic dose could be tolerated by the animals.

### 2.2. Justicidin B Uptake and Bioaccumulation in Short-Term and Chronic Administration

**Figure 3:**
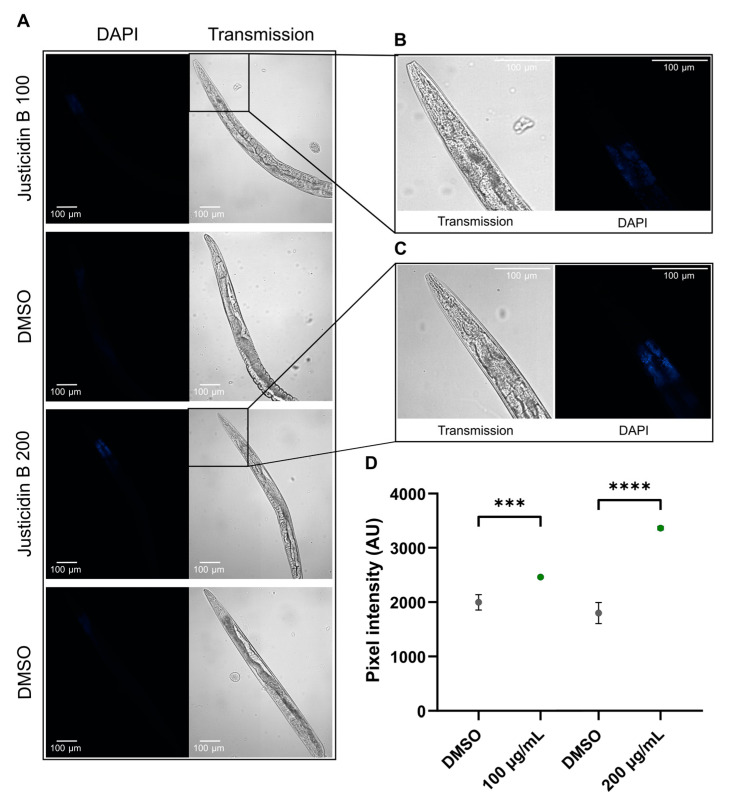
Confocal imaging of *C. elegans* treated with justicidin B in short-term administration. Confocal images of nematodes at the 4th days of justicidin B 100 and 200 µg/mL treatment in short administration were reported. (**A**) DAPI and transmission images of worms treated with Justicidin B and DMSO. (**B**,**C**) Head nematode magnifications both in transmission and DAPI channels of nematodes treated with 100 (**B**) and 200 µg/mL (**C**) of justicidin B. (**D**) Graph representing the quantification of DAPI signals. Data are represented as the mean, and error bars represent the S.E.M. *** *p* < 0.001, **** *p* < 0.0001.

**Figure 4:**
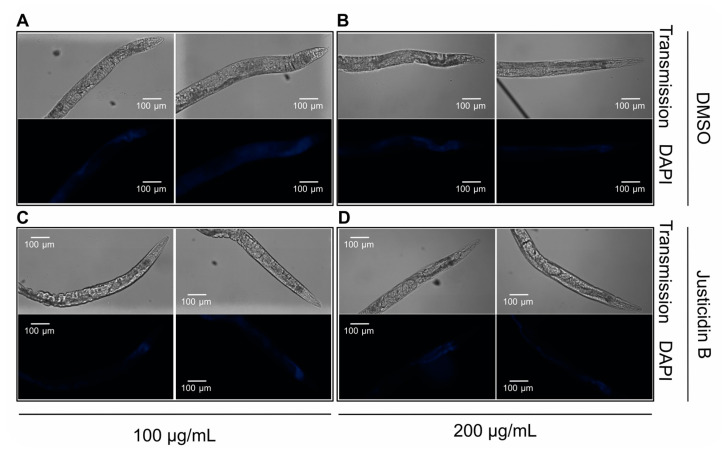
Confocal imaging of *C. elegans* treated with chronic administration of justicidin B. Confocal images, taken at the median day (day 14) of the lifespan, of nematodes treated with justicidin B 100 and 200 µg/mL in chronic administration are shown. (**A**,**B**) DAPI and transmission images of worms treated with DMSO (control). (**C**,**D**) DAPI and transmission images of worms treated with Justicidin B at 100 and 200 µg/mL, respectively.

Since the absorption of a compound could be a limiting step for its bioavailability and toxicity evaluation, justicidin B uptake and distribution in *C. elegans* were analyzed by confocal microscopy (Figure 3). N2 wild-type nematodes treated with short-term administration of 100 and 200 µg/mL were collected on the fourth day of the experiment, washed in PBS and immobilized using NaN3. Justicidin B was visualized with the DAPI channel exploiting its fluorescence [21,22]. The representative images reported in Figure 3A clearly displayed dose-dependent justicidin B bioaccumulation in the first tract of the digestive system (i.e., pharynx, Figure 3B–D). The quantification of DAPI signals showed a statistical difference in comparison with the control (DMSO) of both toxic and non-toxic doses. In particular, we obtained 1.2- and 1.9-fold increases compared to DMSO with 100 and 200 µg/mL, respectively (Figure 3D). Otherwise, confocal imaging obtained on the day corresponding to the median lifespan (fourteenth day for both treated and untreated nematodes, see Table 1) during chronic treatment with 100 and 200 µg/mL did not reveal bioaccumulation of justicidin B in specific parts of the body (Figure 4). In fact, a uniform distribution of fluorescence was observed with both doses, in perfect correlation with the fluorescence of lipofuscin, which was more intense than in the animals treated for short time, owing to its accumulation during aging (Supplementary Figure S1, Figure 4) [23]. It can be argued that justicidin B’s toxicity, shown only in short-term administration, is due to its bioaccumulation.

**Figure 5:**
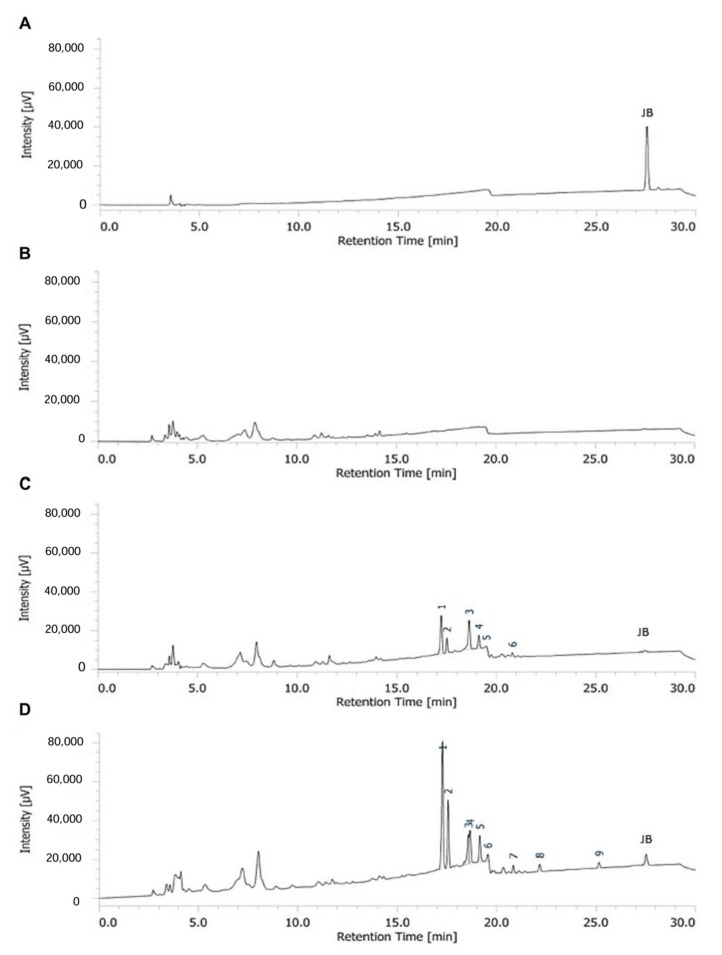
Chromatogram profiles of nematode extracts after short-term treatment with justicidin B (RT = 27.5 min). (**A**) Justicidin B standard (3.125 μg/mL). (**B**–**D**) Nematodes profiles treated with DMSO (**B**), justicidin B at 100 µg/mL (**C**) and justicidin B at 200 µg/mL (**D**). Numbers from one to nine in the chromatogram indicate the metabolic derivatives.

**Figure 6:**
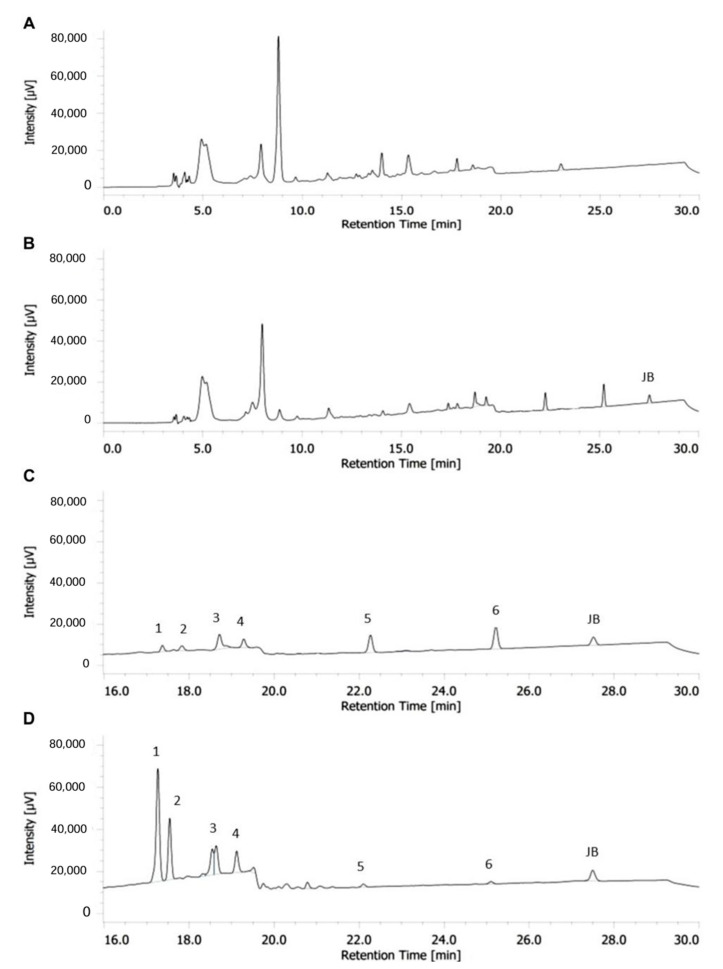
Chromatographic profiles of nematode extracts after chronic treatment with justicidin B (RT = 27.5 min) and comparison with short-term treatment profile. (**A**) Chronic treatment with DMSO (control). (**B**) Chronic treatment with justicidin B at 200 µg/mL. (**C**,**D**) Enlargement of chromatograms from 16 min to 30 min RT of chronic (**C**) and short (**D**) treatment with JB at 200 µg/mL. Numbers from one to six in the chromatogram indicate the metabolic derivatives.

To further validate the hypothesis, the amount of justicidin B contained in the nematode extract obtained at the fourth day of short-term treatment was determined by HPLC analyses. The amount of justicidin B presents in the dry weight of the worms treated with 200 µg/mL was 74.8 ± 14.57 ng/mg and only a trace amount for those grown with 100 µg/mL (Figure 5C,D). Moreover, the HPLC profiles highlighted the presence of other peaks beyond justicidin B that were absent in control samples (Figure 5B). These unknown peaks were present in greater numbers and were more abundant in extracts from nematode treated with 200 µg/mL than 100 µg/mL of justicidin B. These metabolites could represent compounds resulting from justicidin B metabolism. These unknown products are undetectable in growth medium or washing solutions of nematodes both treated and untreated with justicidin B (Figure S2). These results further demonstrated the higher justicidin B accumulation with the 200 µg/mL dose and raised the possibility that the toxicity might also be due to its metabolic derivatives. To pursue this point further, HPLC analysis of worms treated with a 200 µg/mL dose in chronic administration was performed on the fourteenth day. The obtained profile was quite similar to the short-term treatment profile (Figure 5D and Figure 6B), with a comparable amount of justicidin B, but with a significantly lower intensity of peaks 1 and 2 (Figure 6C,D). These differences strongly support the hypothesis that these metabolites could be responsible for justicidin B toxicity.

### 2.3. Justicidin B’s Effect on C. elegans Healthspan Parameters

**Figure 7:**
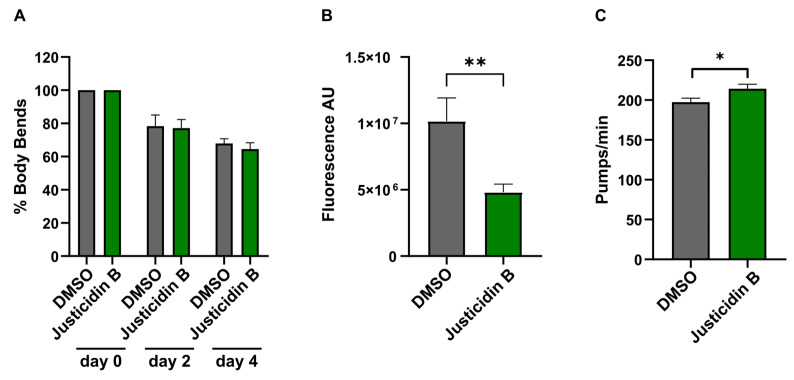
Effects of justicidin B (200 μg/mL) on *C. elegans* healthspan parameters in short-term administration. (**A**) Effect on motility. Body bend counts in one minute are expressed as percentages with respect to day 0. (**B**) Total ROS in *C. elegans* whole organisms at day 4 of administration. ROS signals were obtained using the fluorescent probe DCF-DA. Columns represent the mean of samples’ fluorescent signals, and error bars represent S.E.M. (**C**) Numbers of pharynx contractions in one minute at day 4 of justicidin B administration are reported. Controls were treated with the same DMSO quantity used in the treated samples. Each column represents the mean, and error bars are S.E.M. values of three independent experiments. * *p* < 0.05, ** *p* < 0.01.

To deeper understand the mechanism of justicidin B toxicity in short administration, the effect of 200 µg/mL justicidin B treatment on some physiological parameters, i.e., locomotion, pumping rate and oxidative stress, was evaluated. Motility is considered a measure of neuromuscular function. Therefore, discrepancies in locomotion frequency are symptomatic of dysfunction in specific neuronal or muscle tissues [24]. *C. elegans* movement was quantified by counting the number of body bends per minute both in the presence and in the absence of justicidin B at 200 µg/m. In Figure 7A, the number of body bends was reported after normalization with respect to day 0. No differences in the body bends trend were observed in comparison to the control, excluding a possible justicidin B effect on neuromuscular activity.

*C. elegans* feeding depends on the action of the pharynx, a neuromuscular pump that joins the mouth to the intestine. The rate of pumping determines the amount of food intake and the rate of growth [24]. Moreover, it has been observed that pharyngeal pumping rate decreases in the presence of several known mammalian neurotoxins [25]. The numbers of pumps per minute of 30 nematodes on the fourth day of short-term treatment were quantified, and a slight increase in pump numbers was observed with respect to the worms treated with DMSO (Figure 7B), highlighting that justicidin B did not affect feeding ability. Finally, total ROS production was determined using the permeant probe DCFH-DA [26]. Thirty nematodes from three independent experiments on the fourth day of justicidin B treatment were washed with PBS and incubated at 37 °C in the presence of the probe. The quantitative analysis displayed an approximately twofold reduction in total ROS content in the nematodes treated with Justicidin B (Figure 7C). This result allowed us to exclude oxidative stress as a cause of the toxicity observed with justicidin B at 200 µg/mL.

### 2.4. Justicidin B’s Effect on C. elegans Fertility and Development

**Figure 8:**
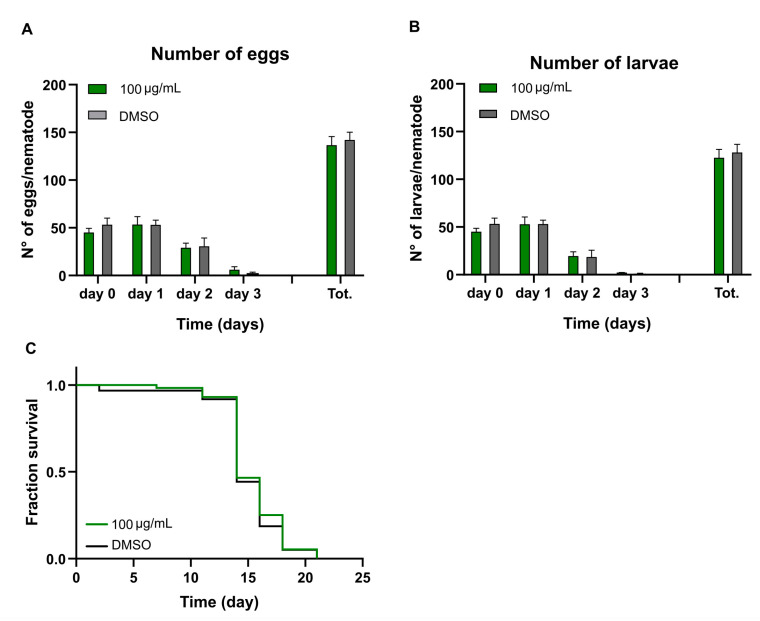
*C. elegans* DART assay for short-term administration of justicidin B (100 μg/mL). Numbers of eggs (**A**) and larvae (**B**) per day produced by nematodes. Each column represents the mean, and error bars are the S.E.M. (**C**) Lifespan assay of F1 progeny derived from nematodes treated with justicidin B. Kaplan–Meyer graph represent the survival of the population. Controls were treated with the same DMSO quantity used in the treated samples.

DART assays in *C. elegans* model take advantage of the 300 progeny produced by each healthy adult worm in 3 days. This, combined with the 3 days of development from larva to adult worm, ensures fast and sensitive detection of toxicity in large-scale populations [24]. Synchronized young adult worms were placed onto NGM plates with and without justicidin B (100 µg/mL) to evaluate the progeny production of the treated group compared to the control (Figure 8). Reproductive toxicity was assessed by calculating the number of offspring for four days in the presence of the compound. No significant alteration in the total fecundity was observed (Figure 8A). In fact, no differences in either egg-laying or the number of larvae obtained from the same eggs were observed (Figure 8B). Finally, the lifespan of the nematodes counted after three days of development of the same larvae displayed the same median and maximum values as the control (Figure 8C and Table 2). All the results clearly demonstrated that the treatment of adult worms with 100 µg/mL justicidin B did not affect either fertility or development.

## 3. Discussion

The continuous evolution of the various forms of cancer with consequent resistance to initially effective therapies, together with the safety problems in the use of chemotherapeutics, makes necessary the continuous research of new anticancer drugs. A promising strategy derives from the screening of plant-derived compounds which display a wide range of biological activity, including anti-tumor activity [1,2]. Nowadays, most pharmaceuticals are plant-derived metabolites, used after extraction from plant material or after chemical semi-synthesis [27]. However, bioactive compound production from plants is very low, reaching approximately 1% of dry matter. In vitro plant cells or tissue cultures represent a good strategy to obtain a higher number of bioactive compounds. This technology allows continuous production independent of plant growth rates and climatic and geographical conditions while avoiding the overexploitation of several endangered species; it is currently used to produce many therapeutics [28,29]. The justicidin B used in this study was produced by adventitious root cultures from *Linum lewisii* [21]. Justicidin B represents a potential lead compound for the development of new anti-cancer therapies thanks to its anti-proliferative and pro-apoptotic activity against a wide range of cancer cell lines [7]. One of the greatest limitations in using a substance as a drug is its bioavailability and its toxicity, especially in long-term therapies. Successful therapy allows therapeutic efficacy to be maintained and side effects reduced. However, toxicity studies in mammalian models are expensive and time-consuming [30] and more than one animal model should be used to obtain predictive human data [31] but this increases the cost and decreases the output. Moreover, the use of alternative methods for toxicity assessments is mandatory in accordance with the 3R requirements. One viable option is the use of small model organisms such as the nematode *C. elegans*, which can be managed using *in vitro* techniques with the advantage of obtaining toxicity data from a whole animal with digestive, reproductive, endocrine, sensory and neuromuscular systems [24]. As an intermediate between cell lines and mammalian models, toxin ranking obtained using various *C. elegans* assays is often predictive for mammalian toxicity [32]. In this work, N2 wild type *C. elegans* has been used to study toxicity of justicidin B treatment. At first, the effect on the worm lifespan of different doses was assayed adding the compound onto the plate for 4 days (short-term treatment) and then in chronic condition. This allowed us to identify the 100 µg/mL as a non-toxic dose administered both daily for a short period and even in a long treatment. On the contrary, the 200 µg/mL dose reduced lifespan but only in short-term treatment (Figure 1 and Figure 2). In the last case, the toxicity could be related by the significant bioaccumulation observed in treated worms through confocal analysis. Indeed, a dose-dependent accumulation of the fluorescence in the first tract of the digestive system was observed (i.e., pharynx, Figure 3B–D). Actually, this fluorescence might be correlated not only to the justicidin B accumulation, but also to the metabolic derivatives revealed by HPLC analysis carried out on the extracts of worms treated with 100 µg/mL and 200 µg/mL in short administration, whose abundance resulted dose dependent. These metabolites were not released into the plate (Figure S2) and therefore may accumulate in the worms. Moreover, as clearly shown by the confocal analysis displayed in the Supplementary Figure S1, the fluorescence signal in the FITC channel, that allows the detection of lipofuscin granules, clearly overlaps with justicidin B or/and metabolites signal (DAPI) (Figure S1). Lipofuscin in *C. elegans* is a non-degradable material obtained by products of fatty acids peroxidation, sugars, metals and oxidized proteins that accumulates over the time in lysosomes of post-mitotic cells (the *C. elegans* entire soma is post-mitotic) resulting in age-dependent degeneration of various cellular systems [33]. Its accumulation seems to be associated with the inability of cellular proteolytic mechanisms to degrade it during aging [33]. It is possible to hypothesize that the hydrophobic nature of justicidin B and/or of its metabolites leads to an accumulation inside lipofuscin granules. It is known that lipophilic drugs tend to have a high affinity for adipose tissue, so they tend to accumulate in body fat both in the case of sporadic use and in particular in the chronic administration. Once they have entered the adipose tissue, the drugs find a balance with their plasma concentration and are eliminated from the body very slowly. This fact implies a prolonged duration of action and the risk of accumulation in the body, with consequent risks of toxicity due to excess of drug, if further intakes are taken before the previous dose has been completely eliminated [34]. Likewise, daily administration with the higher dose of justicidin B led to bioaccumulation that did not occur with a chronic treatment where a new dose is administrated every other day. Thus, less-than-daily treatment at the higher doses is recommended to avoid toxicity (Figure 6). Noteworthy, the comparison between HPLC profiles obtained on worm extract after short (toxic) and chronic (non-toxic) administration clearly supports the hypothesis that justicidin B toxicity could strongly be due to the accumulation of two specific metabolites that are significantly more abundant in the short-term treatment profile (Figure 5 and Figure 6). A further characterization of these two metabolites will be necessary in order to identify their structure and to demonstrate that they are directly responsible for the justicidin B toxicity. The monitoring of healthspan parameters in worms treated in sub-chronic conditions showed that the metabolites accumulation does not induce significant variation in both the movement and pumping rate parameters. Otherwise, a reduction of the total ROS levels was observed, probably due to justicidin B radical scavenging activity [35]. This seems in contrast with previous studies performed in leukemia K562 cells where a reduction of superoxide dismutase (SOD) activity was observed [36] but it could be justified by the different oxidative stress conditions of the cancer cells [37]. Finally, the safety of 100 µg/mL dose was also demonstrated in DART assays. In particular, no significant differences were observed both in the fecundity and in larvae development.

## 4. Materials and Methods

### 4.1. Justicidin B Production and Purification

Justicidin B was obtained from *in vitro* suspension cultures of *Linum lewisii* adventitious roots as described by Dougué Kentsop et al., 2022 [21]. The induction of adventitious roots was performed starting from *L. lewisii* leaves explant grown on MS solid medium supplemented with 3% *w*/*v* sucrose, 0.4 mg/L 1-naphthaleneacetic (NAA) and 1 mg/L 2,4-dichlorophenoxyacetic acid (2,4-D). The root primordia were transferred to an MS medium supplemented with 0.5 mg/L indole-3-butyric acid (IBA) and 0.1 mg/L indole-3-acetic acid (IAA) (MS-II medium), and they were subcultured every month. To obtain enough biomass for the purification of justicidin B, 10 g fresh weight (FW) roots were transferred in 500 mL MS-II liquid medium and grown for five weeks on an orbital shaker at 110 rpm at 25 °C in the dark. The extraction and purification of justicidin B was performed following the method described by Mascheretti et al., 2021 [22]. In detail, a Jasco semi-preparative HPLC system equipped with photodiode array detector was used. The samples were separated through a Synergi polar RP 80 Å column (250 mm × 10 mm, 4 μm, Phenomenex, Torrance, CA, USA) by means of water 0.5% (*v*/*v*) formic acid and acetonitrile 0.5% (*v*/*v*) formic acid as eluents. The purity of the justicidin B obtained was up to 98.80% and was used as standard for HPLC quantification in analytical method.

### 4.2. C. elegans Maintenance and Synchronization

*Caenorhabditis elegans* has been used as a powerful model organism for molecules toxicity studies for its several advantages, including ease of culture, small size, transparent body, short lifespan, well-characterized genome and a high degree of conservation with human genes, including several processes and signaling pathways [16]. The wild-type Bristol *Caenorhabditis elegans* N2 strain was obtained from the Caenorhabditis Genetics Center (CGC, University of Minnesota, Minneapolis, MN, USA). The nematodes were maintained at 20 °C on NGM plates (nematode growth media) seeded with live *Escherichia coli* strain OP50 at OD600 = 0.3 as food source. For the experiments, the worm population was synchronized allowing 10 to 30 gravid adult worms to lay eggs for 16 h at 20 °C on NGM plates seeded with *E. coli* OP50. After this, worms were sacrificed and the plates were kept at 20 °C, until larvae reached the adult phase. 1-day synchronized adult population was used for the experiments.

### 4.3. Justicidin B Treatments

Two different treatments were performed: the first one, called short-term, consisted of administration for four days, starting from the first day of adulthood, of a specific dose of justicidin B; in the second one, defined as chronic, the specific doses were administered every two days until all animals died. Due to its hydrophobic nature (logP = 4), the justicidin B could be dissolved only in organic solvents, as previously reported [7]. DMSO is one of the most used solvent for the delivery of non-polar and poorly water soluble compounds in cell biology for its high biocompatibility and low-toxicity to the cell. In both treatments the different concentrations of justicidin B were added starting from a 1.500 μg/mL justicidin B stock solution in DMSO and the correspondent control was obtained adding the same volume of DMSO. The different concentrations of justicidin B used were diluted in 200 μL final volume of sterile water, spotted on the surface of the NGM plates and let to dry. In the short-term treatment, freshly prepared plates were daily dispensed in the absence of a food source. In details, 30 synchronized nematodes at day 1 of adulthood were placed onto the NGM plates and everyday living nematodes were transferred in a new plate, till the fourth day. In the chronic treatment, 60 synchronized nematodes at day 1 of adulthood were placed onto the NGM plates in the presence of heat-killed food source (*E. coli* strain OP50 OD600 = 0.3) to avoid the possibility that the treatments with justicidin B could directly affect *E. coli* and thus indirectly the nematodes [38] and every two days living worms were transferred in a new plate freshly prepared until all the population died. Nematodes were considered dead if they did not respond to a gentle stimulation with the transfer pick. Three independent experiments were performed. For the short-term treatment experiment, the vitality trend was analyzed using was analyzed using two-way ANOVA test followed by Sidak’s multiple comparisons test correction; the chronic administration was analyzed by monitoring the surviving curves, and *p*-values were obtained using the log-rank test.

### 4.4. Confocal Imaging

Justicidin B is visible in DAPI channel [21,22], and confocal analysis was used to evaluate both localization and accumulation of the molecule. Fifteen nematodes treated in short or chronic administration with 100 and 200 µg/mL of justicidin B or DMSO were collected at the fourth and fourteenth days, respectively; washed in PBS (25 mM potassium phosphate, pH 7.2, 0.15 M NaCl) and immobilized using a solution of 5% glycerol and 1% NaN_3_ in PBS. Images from 20 nematodes per condition were obtained using a Nikon confocal microscope system A1 and processed with Nikon NIS-Elements AR. Both FITC (gain 50 ms) and DAPI (gain 20 ms) filterset were used to selectively identify the signal of justicidin B and lipofuscin aggregates. Justicidin B quantification was performed by the analysis of pixel intensity (AU) using ImageJ 1.54g software and the statistical analysis was performed using the unpaired *t*-test.

### 4.5. HPLC Analysis of Justicidin B Amount from C. elegans Cultures

Dried nematode pellets obtained from cultures at the fourth day of short-term treatment and fourteenth day of chronic treatment were suspended into methanol (6000 μg/mL), crushed with pestle, ultrasonicated for 30 min and shaken overnight at RT. The supernatant obtained after centrifugation at 13,000× *g* for 10 min was used for HPLC analysis following the method described by Mascheretti et al., 2021 [22]. Quantification of justicidin B was performed with a standard calibration curve obtained using seven standard dilutions ranging from 0.019 to 12.5 µg/mL. The linear regression equation was performed by plotting peak areas against injected amounts of justicidin B standard. Calibration curve and the relative data are reported in Supplementary Materials (Figure S3 and Table S1).

### 4.6. C. elegans Healthspan Parameter Evaluation After Short-Term Treatment

For the evaluation of the healthspan parameters, the experiments were performed at the end of the short-term treatment (fourth day). Specifically, for the evaluation of all parameters, 60 age-synchronized nematodes were treated with justicidin B 200 µg/mL as described in Section 2.2 and Reactive Oxygen Species (ROS) levels, pumping rate and body bends were measured as described in the following paragraphs. Control worms were incubated with DMSO.

#### 4.6.1. Body Bend Assay

Thirty worms were used for the motility evaluation through the body bends assay that consists in the measure of the sinusoidal movement performed by nematodes and counted using the SteREO Discovery V12 microscope (Carl Zeiss Microscopy GmbH, Munich, Germany), as previously described [25]. Data from three independent experiments were processed and presented normalizing the number of body bends/min against the control as a percentage of motility. The statistical analysis was performed using 2-way ANOVA followed by Bonferroni’s test.

#### 4.6.2. Pumping Rate Assay

Twenty animals were assessed for the pharyngeal pumping rate assay. The count of the number of pharynx terminal bulb contractions in 1 min was carried out by recording worms using a SteREO Discovery V12 microscope (Carl Zeiss Microscopy GmbH, Germany) and counting the numbers of pumps replaying the movies at one half of the original speed using Windows Movie Maker. The statistical analysis was performed using the unpaired *t*-test.

#### 4.6.3. ROS Measurement

Thirty worms were extensively washed with 100 µL of PBS to remove the excess of justicidin B and transferred into a 96-well plate (30 nematodes in 100 μL PBS). Then, 2,7-dichlorofluorescein diacetate (DCFH-DA; Sigma-Aldrich Co., St. Louis, MO, USA) at the final concentration of 50 μM was added and incubated at 37 °C. DCFH-DA is oxidized by ROS forming the dichlorofluorescein (DCF), a fluorescent product. It is a permeable probe, allowing its use on whole and living nematodes. A microplate reader (Victor 3, PerkinElmer, Waltham, MA, USA) was used to measure the fluorescence (485 nm excitation, 530 nm emission). Three independent experiments were performed, and values were expressed as percentage of fluorescence intensity normalized to the controls. The statistical analysis was performed using the unpaired *t*-test.

### 4.7. Developmental and Reproductive Toxicity (DART) Assays

#### 4.7.1. Fertility Assay

Ten synchronized adult worms were allowed to lay eggs on a 9 cm NGM plate in short-term treatment using justicidin B 100 µg/mL without food source at 20 °C. In detail, each plate contained one worm, and every day the 10 worms were transferred to new plates at the same time until the fourth day of treatment. After the worms were transferred, every plate was examined to count new eggs laid and then incubated at 20 °C for 12 h to determine the number of vital larvae. The statistical analysis was performed using the unpaired *t*-test.

#### 4.7.2. Lifespan Assay on Progeny Obtained from Nematodes Treated with Justicidin B

To assess justicidin B’s effect on larvae development, a lifespan assay was performed as described in Section 2.2 without justicidin B, and using the offspring of synchronized adult nematodes at day 3 of short-term treatment with justicidin B 100 µg/mL. In details, 30 age-synchronized nematodes at day 1 of adulthood were used to perform a short-term treatment (see material and methods, Section 2.3). The eggs laid at day three of the treatment were let to hatch and to become adult on justicidin B 100 µg/mL (F1 population). The synchronized day 1 adult F1 population was used to perform the lifespan assay. Three independent experiments were performed, and *p*-values were obtained using the log-rank test.

**Figure 9:**
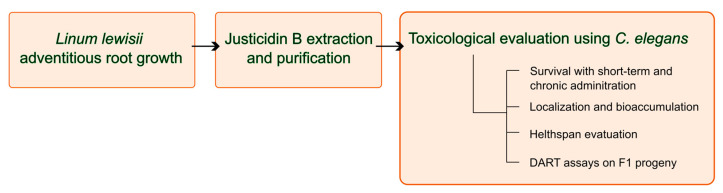
Flowchart of the methodologies used.

A flowchart of all the methodologies used in the manuscript is shown in Figure 9.

### 4.8. Statistical Analysis

All the experiments were replicated at least three times. Statistical analysis and survival curves were performed using GraphPad Prism 8.0.2 software. Kaplan–Meier method was chosen to represent survival curves. This method produces a survival graph over time, which shows the estimated percent survival of the subjects at each point in time. Two-way ANOVA was used in the time point experiments, except for the fertility assay, in which an unpaired *t*-test was chosen. An unpaired t-test was used to compare dose-specific treatment and control group, where the control sample was obtained by adding some volume of DMSO.

## 5. Conclusions

In the present work, the toxicity of the secondary metabolite justicidin B produced by adventitious root cultures of *Linum lewisii* was investigated using a *C. elegans* model. Nematodes proved to be an excellent model for studying toxicity, allowing researchers to acquire significant data in a simplified *in vivo* system. Several areas of concordance with mammals have been widely demonstrated in their toxic responses, which include aging, aneuploidy, growth and development, prediction of mammalian LD50 and neurotoxicity. For predictive toxicology, the *C. elegans* model represents a complementary tool for early toxicity screening and for the identification of conserved modes of toxic action [17]. By taking advantage of this animal model, the short- and long-term safety of 100 µg/mL justicidin B treatment, respectively, have been demonstrated. The treatment, in fact, does not induce any alteration in worms’ healthspan parameters or fecundity. Daily administration of higher doses resulted in toxicity after a short period of treatment, and this is probably due to the pharyngeal accumulation of justicidin B or of two metabolic derivatives. A chemical characterization of these metabolites will be necessary to better understand the mechanisms underlying the toxicity. Moreover, further investigations in mammalian animals will be necessary to confirm the predictivity of our results.

## Figures and Tables

**Table 1:** Lifespan parameters of *C. elegans* treated with chronic administration of justicidin B at 100 and 200 µg/mL. The table presents the median and maximum days of the lifespan reported from the Kaplan–Meyer graph (Figure 2) and expressed as the mean ± standard deviation from three independent experiments.

	Median Lifespan (days) ^1^	Maximum Lifespan (days) ^2^	*p*-Value ^3^
DMSO 100	14.00 ± 0.00	19.50 ± 2.12	n.s
Justicidin B 100	14.25 ± 0.50	20.25 ± 1.50	
DMSO 200	14.00 ± 0.00	22.00 ± 1.41	n.s
Justicidin B 200	13.25 ± 1.15	21.50 ± 1.15	

^1^ Median lifespan was the day when 50% of nematodes used in the assay were alive. Mean ± standard deviation was reported. ^2^ Maximum lifespan was the oldest age reached by the last surviving worm in each treatment. Mean ± standard deviation was reported. ^3^ The *p*-value was calculated using the log-rank test by comparing the control and justicidin B-treated groups. The analysis reveals no significance.

**Table 2:** Lifespan parameters of *C. elegans* F1 population derived from nematodes treated with short-term administration of justicidin B at 100 µg/mL. The median and maximum lifespan in days are reported from the Kaplan–Meyer graph (Figure 7C) and expressed as the mean ± standard deviation from three independent experiments.

	Median Lifespan (days) ^1^	Maximum Lifespan (days) ^2^	*p*-Value ^3^
DMSO	15.00 ± 1.41	23.00 ± 0.00	n.s.
Justicidin B 100	15.33 ± 1.15	21.67 ± 1.15	

^1^ Median lifespan was the day when 50% of nematodes used in the assay were alive. Mean ± standard deviation was reported. ^2^ Maximum lifespan was the oldest age reached by the last surviving worm in each treatment. Mean ± standard deviation was reported. ^3^ The *p*-value was calculated using the log-rank test by comparing the control and justicidin B-treated groups. The analysis reveals no significance.

## Data Availability

The original contributions presented in the study are included in the article and Supplementary Materials; further inquiries can be directed to the corresponding author.

## Supplementary Materials

The following supporting information can be downloaded at: https://www.mdpi.com/article/10.3390/molecules29235516/s1. Figure S1. Confocal images of nematodes at the 14th day of chronic treatment with justicidin B. A and B. Representative images of FITC (A) and DAPI (B) fluorescence of nematode treated with Justicidin B 200 μg/mL in acute administration. (C–F). Representative images of nematodes treated chronically with justicidin B 100 μg/mL (E) and 200 μg/mL (F); (C) and (D) are the relative control nematodes treated with DMSO. Figure S2. Chromatogram profile media and washing solutions at the end of 4th day of growth with and without justicidin B treatments. (A) Washing solution of nematode without justicidin B. (B) Growth medium of nematode without justicidin B. (C) Washing solution of nematode with justicidin B 200 µg/mL. (D) Growth medium of nematode with justicidin B 200 μg/mL and (E) Standard solution of justicidin B 12.5 μg/mL. Figure S3. Calibration curve of justicidin B standard (from 0.098 to 12.5 µg/mL). Table S1. HPLC calibration curve data.

## References

[1] Balunas M.J., Kinghorn A.D. (2005). Drug Discovery from Medicinal Plants. Life Sci..

[2] Elshafie H.S., Camele I., Mohamed A.A. (2023). A Comprehensive Review on the Biological, Agricultural and Pharmaceutical Properties of Secondary Metabolites Based-Plant Origin. Int. J. Mol. Sci..

[3] Savithramma N., Rao M.L., Ankanna S. (2011). Screening of Traditional Medicinal Plants for Secondary Metabolites. Int. J. Res. Pharm. Sci..

[4] Ionkova I. (2011). Anticancer Lignans--from Discovery to Biotechnology. Mini Rev. Med. Chem..

[5] DellaGreca M. (2013). Isolation of Lignans as Seed Germination and Plant Growth Inhibitors from Mediterranean Plants and Chemical Synthesis of Some Analogues. Phytochem. Rev..

[6] Gertsch J., Tobler R.T., Brun R., Sticher O., Heilmann J. (2003). Antifungal, Antiprotozoal, Cytotoxic and Piscicidal Properties of Justicidin B and a New Arylnaphthalide Lignan from *Phyllanthus piscatorum*. Planta Med..

[7] Hemmati S., Seradj H. (2016). Justicidin B: A Promising Bioactive Lignan. Molecules.

[8] Lievens D., von Hundelshausen P. (2011). Platelets in Atherosclerosis. Thromb. Haemost..

[9] Rao Y.K., Fang S.-H., Tzeng Y.-M. (2006). Anti-Inflammatory Activities of Constituents Isolated from *Phyllanthus polyphyllus*. J. Ethnopharmacol..

[10] Momekov G., Yossifov D., Guenova M., Michova A., Stoyanov N., Konstantinov S., Ionkov T., Sacheva P., Ionkova I. (2014). Apoptotic Mechanisms of the Biotechnologically Produced Arylnaphtalene Lignan Justicidin B in the Acute Myeloid Leukemia-Derived Cell Line HL-60. Pharmacol. Rep..

[11] Momekov G., Konstantinov S., Dineva I., Ionkova I. (2011). Effect of Justicidin B—A Potent Cytotoxic and pro-Apoptotic Arylnaphtalene Lignan on Human Breast Cancer-Derived Cell Lines. Neoplasma.

[12] Jin H., Yin H.-L., Liu S.-J., Chen L., Tian Y., Li B., Wang Q., Dong J.-X. (2014). Cytotoxic Activity of Lignans from *Justicia procumbens*. Fitoterapia.

[13] Tajuddeen N., Muyisa S., Maneenet J., Nguyen H.H., Naidoo-Maharaj D., Maharaj V., Awale S., Bringmann G. (2024). Justicidin B and Related Lignans from Two South African *Monsonia* Species with Potent Activity against HeLa Cervical Cancer Cells. Phytochem. Lett..

[14] Ilieva Y., Zhelezova I., Atanasova T., Zaharieva M.M., Sasheva P., Ionkova I., Konstantinov S. (2014). Cytotoxic Effect of the Biotechnologically-Derived Justicidin B on Human Lymphoma Cells. Biotechnol. Lett..

[15] Al-Qathama A., Gibbons S., Prieto J.M. (2017). Differential Modulation of Bax/Bcl-2 Ratio and Onset of Caspase-3/7 Activation Induced by Derivatives of Justicidin B in Human Melanoma Cells A375. Oncotarget.

[16] Girard L.R., Fiedler T.J., Harris T.W., Carvalho F., Antoshechkin I., Han M., Sternberg P.W., Stein L.D., Chalfie M. (2007). WormBook: The Online Review of *Caenorhabditis elegans* Biology. Nucleic Acids Res..

[17] Hunt P.R. (2017). The C. Elegans Model in Toxicity Testing. J. Appl. Toxicol..

[18] Xiong H., Pears C., Woollard A. (2017). An Enhanced C. Elegans Based Platform for Toxicity Assessment. Sci. Rep..

[19] Harlow P.H., Perry S.J., Widdison S., Daniels S., Bondo E., Lamberth C., Currie R.A., Flemming A.J. (2016). The Nematode *Caenorhabditis elegans* as a Tool to Predict Chemical Activity on Mammalian Development and Identify Mechanisms Influencing Toxicological Outcome. Sci. Rep..

[20] Boyd W.A., Smith M.V., Co C.A., Pirone J.R., Rice J.R., Shockley K.R., Freedman J.H. (2016). Developmental Effects of the ToxCast^TM^ Phase I and Phase II Chemicals in *Caenorhabditis elegans* and Corresponding Responses in Zebrafish, Rats, and Rabbits. Environ. Health Perspect..

[21] Dougué Kentsop R.A., Consonni R., Alfieri M., Laura M., Ottolina G., Mascheretti I., Mattana M. (2022). *Linum lewisii* Adventitious and Hairy-Roots Cultures as Lignan Plant Factories. Antioxidants.

[22] Mascheretti I., Alfieri M., Lauria M., Locatelli F., Consonni R., Cusano E., Dougué Kentsop R.A., Laura M., Ottolina G., Faoro F. (2021). New Insight into Justicidin B Pathway and Production in *Linum austriacum*. Int. J. Mol. Sci..

[23] Pincus Z., Mazer T.C., Slack F.J. (2016). Autofluorescence as a Measure of Senescence in *C. elegans*: Look to Red, Not Blue or Green. Aging.

[24] Corsi A.K., Wightman B., Chalfie M. (2015). A Transparent Window into Biology: A Primer on *Caenorhabditis elegans*. WormBook.

[25] Boyd W.A., McBride S.J., Freedman J.H. (2007). Effects of Genetic Mutations and Chemical Exposures on *Caenorhabditis elegans* Feeding: Evaluation of a Novel, High-Throughput Screening Assay. PLoS ONE.

[26] Yoon D.S., Lee M.-H., Cha D.S. (2018). Measurement of Intracellular ROS in *Caenorhabditis elegans* Using 2′,7′-Dichlorodihydrofluorescein Diacetate. Bio Protoc..

[27] Espinosa-Leal C.A., Puente-Garza C.A., García-Lara S. (2018). In Vitro Plant Tissue Culture: Means for Production of Biological Active Compounds. Planta.

[28] Chandran H., Meena M., Barupal T., Sharma K. (2020). Plant Tissue Culture as a Perpetual Source for Production of Industrially Important Bioactive Compounds. Biotechnol. Rep..

[29] Dehelean C.A., Marcovici I., Soica C., Mioc M., Coricovac D., Iurciuc S., Cretu O.M., Pinzaru I. (2021). Plant-Derived Anticancer Compounds as New Perspectives in Drug Discovery and Alternative Therapy. Molecules.

[30] Tralau T., Riebeling C., Pirow R., Oelgeschläger M., Seiler A., Liebsch M., Luch A. (2012). Wind of Change Challenges Toxicological Regulators. Environ. Health Perspect..

[31] Olson H., Betton G., Robinson D., Thomas K., Monro A., Kolaja G., Lilly P., Sanders J., Sipes G., Bracken W. (2000). Concordance of the Toxicity of Pharmaceuticals in Humans and in Animals. Regul. Toxicol. Pharmacol..

[32] Ferguson M., Boyer M., Sprando R. (2010). A Method for Ranking Compounds Based on Their Relative Toxicity Using Neural Networking, *C. elegans*, Axenic Liquid Culture, and the COPAS Parameters TOF and EXT. Open Access Bioinform..

[33] Papaevgeniou N., Hoehn A., Grune T., Chondrogianni N. (2017). Lipofuscin Effects in *Caenorhabditis elegans* Ageing Model. Free Radic. Biol. Med..

[34] Markovic M., Ben-Shabat S., Aponick A., Zimmermann E.M., Dahan A. (2020). Lipids and Lipid-Processing Pathways in Drug Delivery and Therapeutics. Int. J. Mol. Sci..

[35] Rao Y.K., Geethangili M., Fang S.-H., Tzeng Y.-M. (2007). Antioxidant and Cytotoxic Activities of Naturally Occurring Phenolic and Related Compounds: A Comparative Study. Food Chem. Toxicol..

[36] Luo J., Hu Y., Kong W., Yang M. (2014). Evaluation and Structure-Activity Relationship Analysis of a New Series of *Arylnaphthalene lignans* as Potential Anti-Tumor Agents. PLoS ONE.

[37] Hayes J.D., Dinkova-Kostova A.T., Tew K.D. (2020). Oxidative Stress in Cancer. Cancer Cell.

[38] Liao V.H.-C., Yu C.-W., Chu Y.-J., Li W.-H., Hsieh Y.-C., Wang T.-T. (2011). Curcumin-Mediated Lifespan Extension in *Caenorhabditis elegans*. Mech. Ageing Dev..

