# Supplementary material for: Toxicological Analysis of the Arylnaphthalene Lignan Justicidin B Using a Caenorhabditis elegans Model
Source: Molecules. 2024 Nov 22;29(23):5516. doi: 10.3390/molecules29235516 (PMC11643176; doi:10.3390/molecules29235516)
Supplement: Supplementary file 1 [file molecules-29-05516-s001.zip › molecules-3296110-supplementary.pdf]

Supplementary Figure and table Sciandrone manuscript

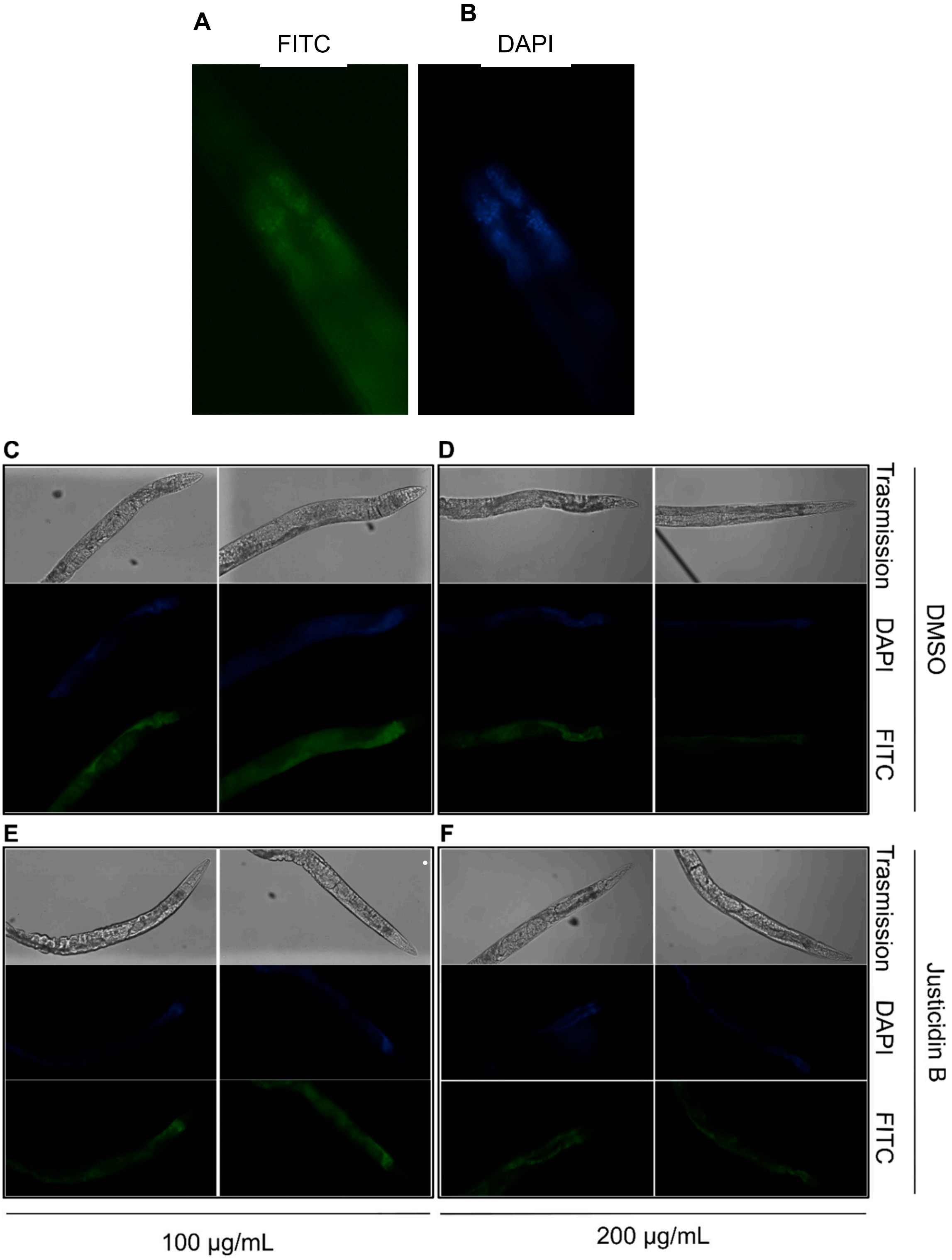

Figure S1. Confocal images of nematodes at the 14th day of chronic treatment with justicidin B. A and B. Representative images of FITC (A) and DAPI (B) fluorescence of nematode treated with Justicidin B 200 µg/mL in acute administration. C, D, E and F. Representative images of nematodes treated chronically with justicidin B 100 µg/mL (E) and 200 µg/mL (F); C and D are the relative control nematodes treated with DMSO.

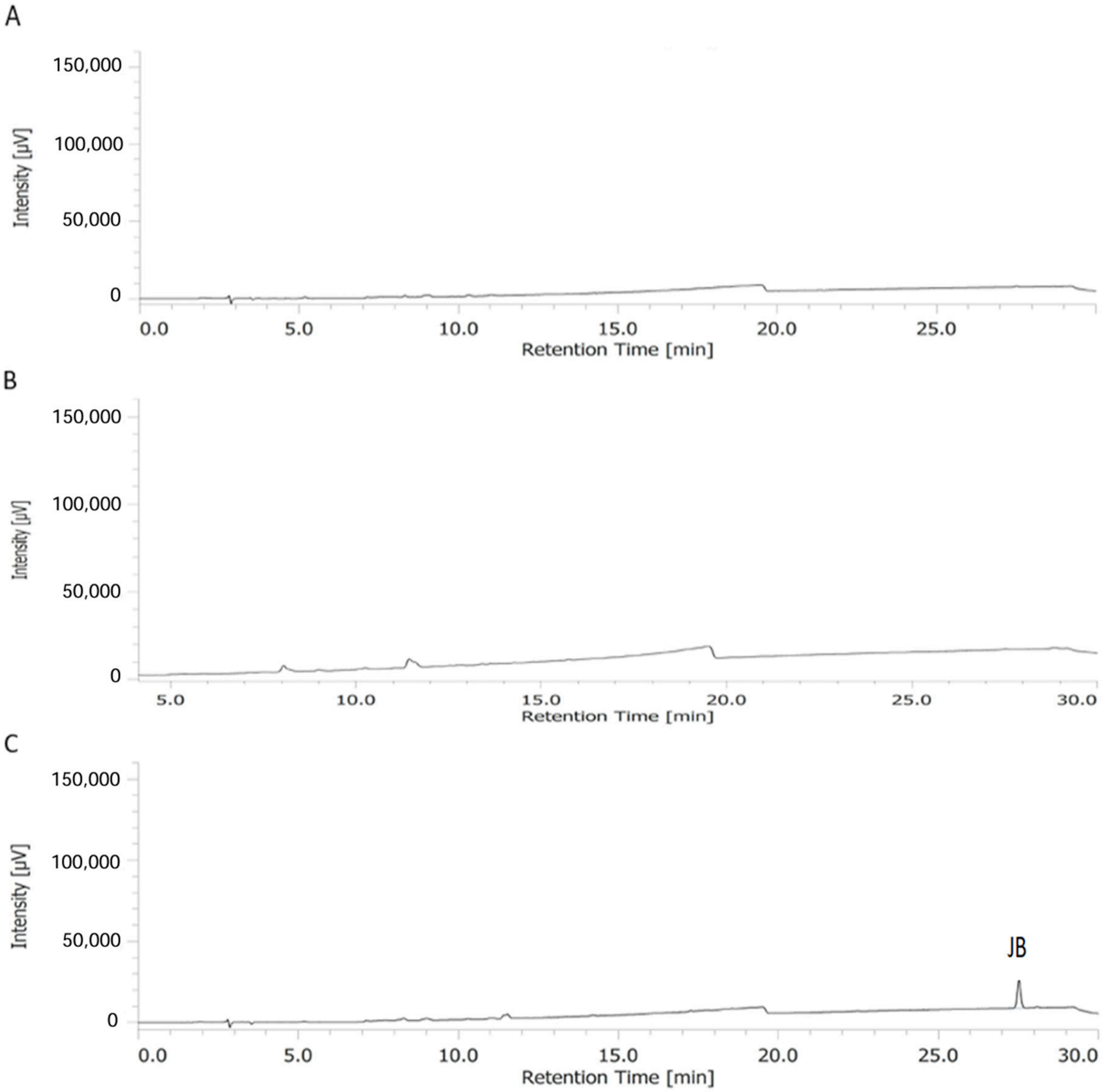

A
150,000
100,000
50,000
0
Intensity [µV]
0.0
5.0
10.0
15.0
20.0
25.0
Retention Time [min]
B
150,000
100,000
50,000
0
Intensity [µV]
5.0
10.0
15.0
20.0
25.0
30.0
Retention Time [min]
C
150,000
100,000
50,000
0
Intensity [µV]
JB
0.0
5.0
10.0
15.0
20.0
25.0
30.0
Retention Time [min]

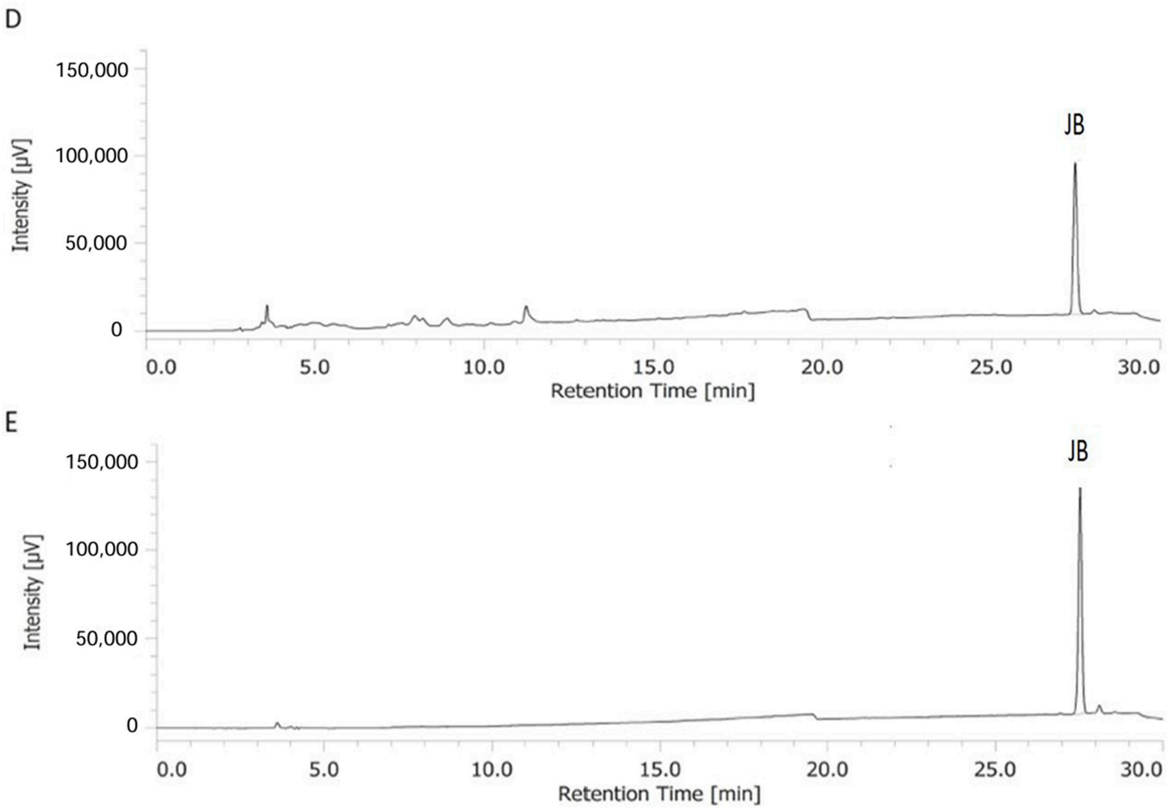

Figure S2. Chromatogram profile media and washing solutions at the end of 4th day of growth with and without justicidin B treatments. A) Washing solution of nematode without justicidin B. B) Growth medium of nematode without justicidin B. C) Washing solution of nematode with justicidin B 200 µg/mL. D) Growth medium of nematode with justicidin B 200 µg/mL and (E) Standard solution of justicidin B 12.5 µg/mL.

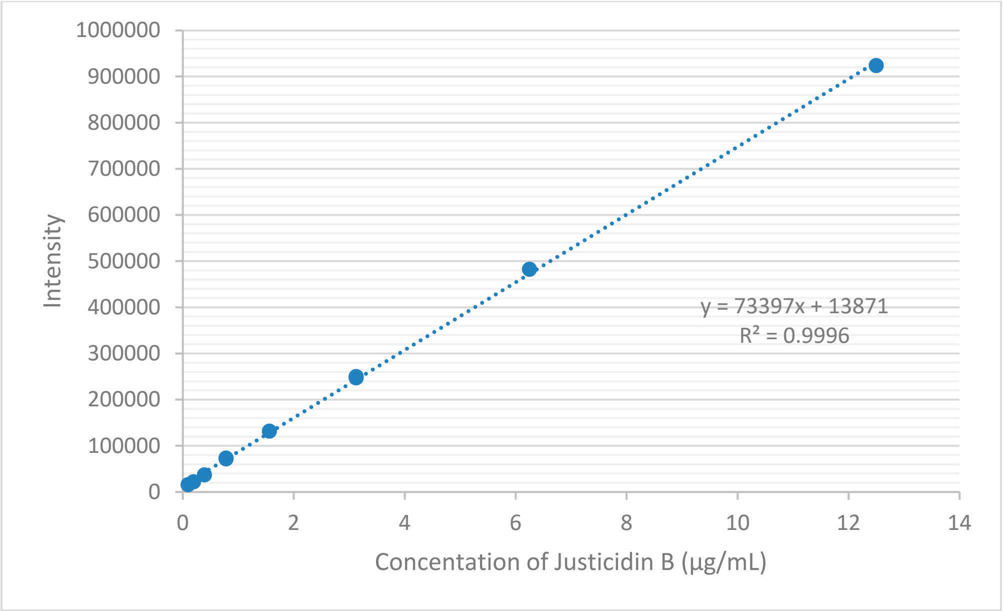

Figure S3. Calibration curve of justicidin B standard (from 0,098 to 12,5 µg/mL).

Table S1. HPLC calibration curve data.

Standard	Concentration range (μg/mL)	Regression equation	Correlation Coefficient (R^2)	LOD (μg/mL)	LOQ (μg/mL)
**justicidin B**	**0,098-12,5**	**y = 73397x + 13871**	**0,9996**	**0,12**	**0,35**
